# Genomic Diversity and Chromosomal Rearrangements in *Neisseria gonorrhoeae* and *Neisseria meningitidis*

**DOI:** 10.3390/ijms232415644

**Published:** 2022-12-09

**Authors:** Boris Shaskolskiy, Dmitry Kravtsov, Ilya Kandinov, Ekaterina Dementieva, Dmitry Gryadunov

**Affiliations:** Center for Precision Genome Editing and Genetic Technologies for Biomedicine, Engelhardt Institute of Molecular Biology, Russian Academy of Sciences, 119991 Moscow, Russia

**Keywords:** *Neisseria gonorrhoeae*, *Neisseria meningitidis*, whole genome sequencing, chromosomal rearrangements, mobile elements

## Abstract

Chromosomal rearrangements in *N. gonorrhoeae* and *N. meningitidis* were studied with the determination of mobile elements and their role in rearrangements. The results of whole-genome sequencing and de novo genome assembly for 50 *N. gonorrhoeae isolates* collected in Russia were compared with 96 genomes of *N. gonorrhoeae* and 138 genomes of *N. meningitidis* from the databases. Rearrangement events with the determination of the coordinates of syntenic blocks were analyzed using the SibeliaZ software v.1.2.5, the minimum number of events that allow one genome to pass into another was calculated using the DCJ–indel model using the UniMoG program v.1.0. Population-level analysis revealed a stronger correlation between changes in the gene order and phylogenetic proximity for *N. meningitidis* in contrast to *N. gonorrhoeae*. Mobile elements were identified, including Correa elements; Spencer-Smith elements (in *N. gonorrhoeae*); Neisserial intergenic mosaic elements; IS elements of IS5, IS30, IS110, IS1595 groups; Nf1–Nf3 prophages; NgoФ1–NgoФ9 prophages; and Mu-like prophages Pnm1, Pnm2, MuMenB (in *N. meningitidis*). More than 44% of the observed rearrangements most likely occurred with the participation of mobile elements, including prophages. No differences were found between the Russian and global *N. gonorrhoeae* population both in terms of rearrangement events and in the number of transposable elements in genomes.

## 1. Introduction

A considerable part of modern approaches in molecular biology are based on whole genome sequencing (WGS) technologies. The results of WGS and de novo genome assembly showed great variability in the structure of genomes even in closely related organisms. Genome rearrangements are characteristic of both eukaryotic and prokaryotic organisms [1] and changes in the order of genes can lead to changes in the properties of a microorganism [2,3]. Studies of the evolution of prokaryotes have shown that the order of genes in prokaryotes is relatively poorly preserved in the process of evolution and, as a rule, changes faster than their amino acid sequences [2,3,4,5].

The comparison of genome sequences of closely related species reveals the role of genomic rearrangements in the formation of macroscopic polymorphisms through various recombination events: insertions, deletions, inversions, and translocations. The frequency of observed genomic rearrangements correlates with the number of mobile genetic elements and the state of repair/recombination systems in genomes [5,6].

The genus *Neisseria* includes two species of bacteria pathogenic to humans: *N. gonorrhoeae* and *N. meningitidis*, the causative agents of gonorrhea and meningococcal infection, respectively. Unlike gonococcus, meningococcus can be present in the human nasopharyngeal mucosa without causing invasive disease.

As in other bacteria, changes in the order of genes in bacteria of the *Neisseria* genus can occur as a result of recombination which is divided into homologous, site-specific, and illegitimate. Homologous recombination is an exchange of aligned regions of homologous DNAs, and its frequency is determined primarily by the extent of homology. Site-specific recombination occurs between specific sequences with short homologous regions. Illegitimate recombination involves DNA sequences without homology. In *Neisseria*, as in many other species, the RecA protein, which has multiple activities related to DNA recombination and repair, plays the central role in the process of homologous recombination. At the same time, *N. gonorrhoeae* has both RecBCD and RecF DNA repair pathways [7].

During the evolution of *Neisseria* spp., numerous chromosomal rearrangements occur, and the structural elements which are most often involved in rearrangements are various repetitive sequences and prophages. The most important repetitive sequences include Neisserial intergenic mosaic elements (NIMEs), Correia repeat enclosed element (CREE), and various insertion sequences (ISs).

NIMEs with a length of 70–200 bp include 50–150 bp repetitive sequences (RS elements) flanked by 20 bp inverted repeats ATTCCC(N)_8_GGGAAT called duplicated repeat sequences (dRS3). dRS3 are present in about 200 copies in the *N. gonorrhoeae* genome and up to 700 copies in *N. meningitidis* and are the second most common repetitive sequences after the DNA uptake sequence (DUS). In addition to flanking RS elements, dRS3 can act as sites for phage integration [8,9,10,11,12].

Correia elements consist of a characteristic core region that defines their length (104–108 or 153–157 bp), surrounded by 26 bp inverted repeats called Correia repeats, and may carry promoter sequences at their ends at position −10 and/or −35 (Black promoter, Snyder promoter). Some longer CREEs carry a recognition site for integration host factor (IHF) that affects the expression of neighboring genes [13,14,15]. *N. gonorrhoeae* contains 120–150 CREE copies dispersed throughout the genome [14,15], while *N. meningitidis* contains twice as many copies, about 250 [8]. Commensal *Neisseria*, with the exception of *N. sicca*, have a lower number of CREEs compared to pathogenic ones [10].

*N. gonorrhoeae* elements about 650 bp in length containing 19 bp inverted repeats 5′-CGTTTCAGACGGCATCGGG//CCCGATGCCGCCTGAAACG-3′ were first described in [16]. These elements named Spencer-Smith Repeat Enclosed Elements (SSREEs), three elements in each strain, were found in different strains of *N. gonorrhoeae* at approximately the same positions in the genomic sequences. The inner SSREE segment has open reading frames, but they do not encode transposases.

Bacteria also contain various ISs, mobile DNA fragments 700–2500 bp in length. IS elements have one or two open reading frames encoding proteins responsible for functions involved in their motility (transposases). On the right and left sides, ISs are bounded by short terminal inverted repeats (IRs) [17]. IS elements are grouped into families, depending primarily on the type of transposase. The IS30 elements encode the DDE-type transposase (with a conserved amino acid triad, Asp-Asp-Glu, in the active site) working on the “copy-and-paste” principle. The IS110 and IS1595 elements encode a DEDD (Asp-Glu-Asp-Asp)-type transposase. Although the chemical mechanisms of action of DEDD and DDE enzymes are similar, the mechanisms of operation of IS30 and IS110 elements are presumably different [18,19]. It was shown that the IS1655 element (IS30 group) is restricted only to *N. meningitidis* [20].

To describe horizontal transfer and integration of genes, the model of minimal mobile elements (MMEs) was proposed [21]. MMEs are cassettes surrounded by conservative genes encoding proteins, the content of which differs between strains or species. Cassettes can be incorporated into the genome by homologous recombination. Comparative analysis of *Neisseria* genome sequences revealed more than 30 potential MME sites, many of which contain strain-specific genes that may have been acquired through horizontal transfer even from other genera of bacteria [21].

“Hot spots” of recombination are often associated with the locations of prophages, i.e., sets of phage genes integrated into chromosomal DNA. Prophage elements can include modules of genes for regulation, transposition, lysis, as well as phage head and tail proteins.

In the study of the *N. gonorrhoeae* FA1090 genome sequence, five bacteriophages with double-stranded DNA (NgoΦ1–NgoΦ5) and four filamentous phages (NgoΦ6–NgoΦ9) have been identified [22,23]. Phages NgoΦ1 and NgoΦ2 have all the genes necessary for functioning; the rest of the double-stranded phages are incomplete. All four single-stranded phages are structurally similar and are associated with IS-like elements ISNgo2 and ISNgo3. It is believed that phages integrate into the genome using their own transposases. Neisserial genome possesses intact copies of prophages, truncated variants, variants with internal deletions, and inverted sequences.

Meningococci do not have sequences of double-stranded phages present in *N. gonorrhoeae*; instead, they have Mu-like phages MuMenB and PNM1-PNM2 which are absent in *N. gonorrhoeae* [12,22]. The Mu phage is a mobile element encoding its own transposase, more traditional phage proteins and a series of unique DNA and protein elements [24]. Its distinguishing feature is the ability to integrate its DNA into an extremely large number of regions of the host genome [25]. Importantly, it induces transfer of resistance genes among bacteria during the phage lytic cycle when bacterial resistance genes are packaged into the Mu phage [26]. Mu-like prophages have been found in many bacterial species, indicating their widespread use as mobile genetic elements [12,22,24].

By now the analysis of genomic rearrangements was described for several strains for which WGS data were available at the time of the study. The analysis mainly concerned rearrangements in two pathogenic *Neisseria* species [8,9,13,14,16,27], but in several studies a comparative whole genome analysis of pathogenic and commensal *Neisseria* was carried out [10,28].

Analysis of three *N. gonorrhoeae* strains (FA1090, NCCP11945, and TCDC-NG08107), the complete genome sequences of which were determined in 2012, showed 14 breakpoints in chromosomal synteny that were mainly associated with a prophage, IS elements, or IS-like repeat enclosed elements. It was shown that the gonococcal genome contained at least 16 IS1016 elements. IS1016 were found at both ends of DNA fragments that turned out to be inverted, indicating that these ISs are associated with large-scale chromosomal rearrangements. Three SSREEs contributed to the appearance of inversions in strain FA1090, being a template for homologous recombination [16].

To determine the time frame during which changes in the order of genes can occur, the rearrangements in *N. gonorrhoeae* strains exposed to stress factors ((39–41 °C), nalidixic acid) were determined in laboratory conditions [15,29]. Structural rearrangements associated with CREEs and prophage-associated elements appeared after 8 weeks of growing the culture under stress and reseeding every three days.

*N. meningitidis* has a highly flexible and variable genome [20,27]. Rearrangements in *N. meningitidis* were studied mainly in terms of their effect on the virulence of strains. Whole-genome comparison of disease and carriage strains (seven strains in total) revealed the important role of Nf1 filamentous prophage in chromosomal rearrangements and translocation of some candidate virulence genes [27].

Recently, there has been a significant increase in information about the structure of genomes based on WGS using long reads, which makes it possible to reliably determine the order of genes on a chromosome. In this work, we studied rearrangements in the genomes of *N. gonorrhoeae* and *N. meningitidis* on the basis de novo sequencing data available from databases and *N. gonorrhoeae* genomes sequenced in our laboratory. The aim of this work was to determine changes in the order of genes with the analysis of the reasons for the transition to the current gene order, to find mobile elements and to establish their role in rearrangements. It was also interesting to compare rearrangements in these two bacterial species and to assess the dependence of gene order on phylogenetic proximity.

## 2. Results

### 2.1. Whole Genome Sequencing of N. gonorrhoeae Isolates Collected in Russia

The results of WGS and genome assembly of 50 Russian *N. gonorrhoeae* isolates showed the presence of a 2.2 million bp circular chromosome in all strains. In all samples, 4207 bp cryptic plasmids were identified. The isolates belonged to the G807 (NG-MAST 228, 807, 1544, 5941, 9570, 9576, 13054), G1993 (NG-MAST 1993, 5714, 14006, 14018) and G14942 (NG-MAST 9476, 9842, 14942) genogroups common in the Russian Federation [30,31]. The MLST typing showed the presence of eight MLST types: 1594, 1901, 11177, 13759, 14009, 14013, 15101, and 15664. A list of isolates and their characteristics are given in Table S1 of the Supplementary Materials.

Combining genome sequences generated using two different WGS platforms at the data processing stage allowed us to obtain a high density of overlapping reads in the final assembly (hybrid assembly), which made it possible to process data without using a reference genome. De novo genome assembly using long reads enabled us to obtain data on the order of genes on the *N. gonorrhoeae* chromosome and to carry out an analysis of genomic rearrangements. As a control, we sequenced the DNA of the *N. gonorrhoeae* strain ATCC 49226 (Bioproject PRJNA768989, SAMN28870224) in our laboratory and compared the results with the sequence of this strain from the database. Nucleotide sequences were 99.97% identical without any changes in gene order.

### 2.2. Neisseria Isolates from the Databases

The GenBank and BV-BRC databases were searched for the genome sequences that were obtained by de novo assembly with high coverage. A total of *96 N. gonorrhoeae* and 138 *N. meningitidis* genomes were found and used in this work to study chromosomal rearrangements.

The sample of *N. gonorrhoeae* genomes consisted of 50 genomes of isolates collected in Russia and sequenced in this work and 96 genomes of isolates collected worldwide and available from public databases, including 15 WHO genomes, to give a total of 146 genomes. The sample of *N. meningitidis* genomes derived from 138 isolates contained genomes from 26 countries, including countries of the African meningitis belt (26 sub-Saharan countries from Senegal to Ethiopia) (Table S1).

### 2.3. Phylogenetic Network of N. gonorrhoeae and N. meningitidis Isolates

*N. gonorrhoeae* isolates belonged to 45 different MLST types, the most common of which were 1594, 7363 and 1901. *N. meningitidis* isolates belonged to 38 MLST types, the most common of which were MLST 11, 23, 2881.

**Figure 1:**
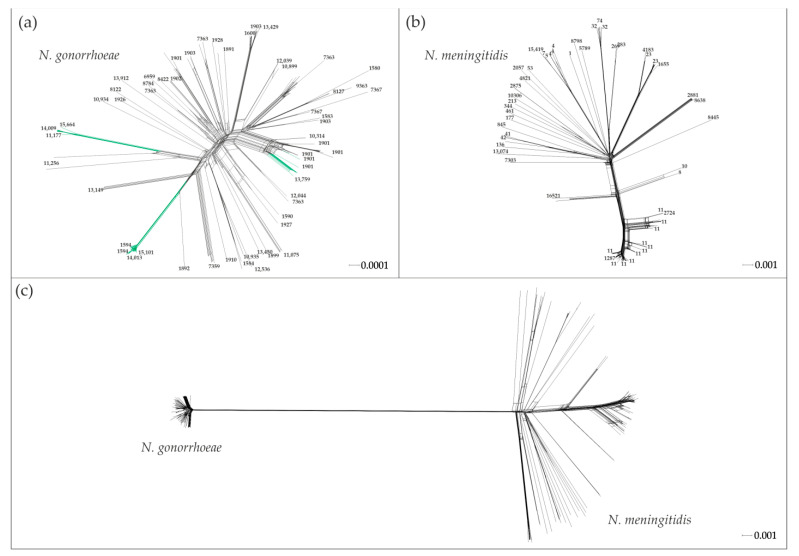
Phylogenetic network based on core genomes for isolates of *N. gonorrhoeae* (**a**), *N. meningitidis* (**b**), together for *N. gonorrhoeae* and *N. meningitidis* (**c**). Numbers denote MLST types. Genomes of the Russian *N. gonorrhoeae* isolates are colored in green.

Based on the obtained sequences of the core genomes of *N. gonorrhoeae* and *N. meningitidis* isolates, we constructed a phylogenetic network that characterizes the relationship between isolates of both species (Figure 1a–c). As can be seen from Figure 1c, the genetic diversity of *N. gonorrhoeae* and *N. meningitidis* isolates differs significantly: the population of *N. meningitidis* is characterized by a greater diversity.

The measure of genome diversity can be characterized by the value of π which determines the average number of pairwise nucleotide differences among genome sequences [32]. The calculated value of π for *N. gonorrhoeae* (2.9 × 10^–3^) was about six times less than that for *N. meningitidis* (17.3 × 10^–3^).

### 2.4. Estimation of the Number of Rearrangement Events in Genomes of N. gonorrhoeae and N. meningitidis

Syntenic blocks and their locations in *N. gonorrhoeae* and *N. meningitidis* genomes were determined. In 146 *N. gonorrhoeae* genomes, 220 syntenic blocks were identified. The number of common blocks, i.e., blocks found in all analyzed genomes, was 51. The total number of blocks per genome varied from 65 to 86, averaging 75. For 138 *N. meningitidis* genomes, 327 syntenic blocks were identified. The number of common blocks found in all analyzed genomes was 126. The total number of blocks per genome varied from 158 to 186, averaging 175. The list of identified syntenic blocks for *N. gonorrhoeae* and *N. meningitidis* is given in Table S2.

**Figure 2:**
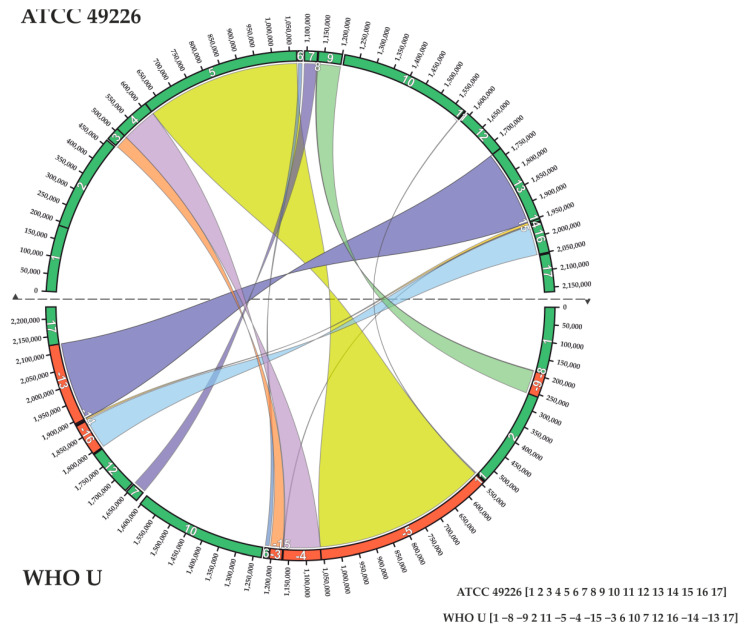
Representation of rearrangements between genomes and calculation of the minimum number of rearrangement events on the example of two *N. gonorrhoeae genomes*, ATCC 49226 and WHO U. The results of pairwise comparison of genomes are presented as a string with the numbers of syntenic blocks and their order; the minus sign means a change in the direction of the genes (inversion). Blocks are highlighted in different colors; block numbers are marked with white numbers on the pie chart. Orange color reflects a change in the strand direction. The minimum number of rearrangement events that allow one genome to turn into another (DCJ distance) in this case is equal to 11.

Rearrangement events and calculation of the DCJ–indel distance by the example of two *N. gonorrhoeae* genomes, ATCC 49226 and WHO U, are illustrated in Figure 2.

Next, we identified the minimum number of changes in the genome structure that allow a genome with one order of syntenic blocks to pass into a genome with a different order of syntenic blocks (the number of genome rearrangements). For the studied sample of *N. gonorrhoeae* genomes, the number of changes in genomes varied from 0 to 28; for *N. meningitidis* genomes, from 0 to 43, with the minimum length of syntenic block set at 1248 bp. It should be noted that the number of rearrangement events in the studied *N. gonorrhoeae* population did not depend on the NG-MAST type and the MLST type of isolates, while in the *N. meningitidis* population, it did not depend on the MLST type and the serogroup.

We examined the possibility of genome changes during the experiment, i.e., the possibility of the appearance of rearrangements during various manipulations with cell cultures. To do this, two *N. gonorrhoeae* DNA samples from isolates obtained in Russia were re-sequenced and re-analyzed (Bioproject PRJNA768989, SAMN31014989, SAMN22599488, SAMN31015000 and SAMN22600865). The analysis showed that, at least during the course of the experiment, the sequencing results were identical and no changes in synteny were observed.

**Figure 3:**
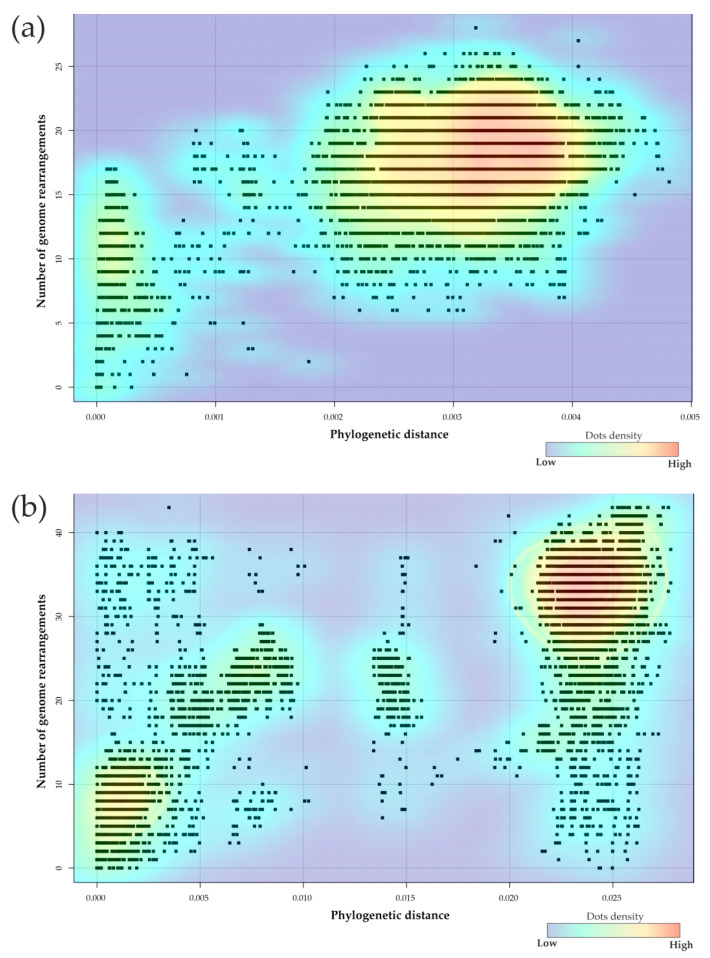
Relationship between phylogenetic distance estimated as the number of base substitutions per site between sequences in core genomes and the number of genome rearrangements for *N. gonorrhoeae* (**a**) and *N. meningitidis* (**b**).

Then, we analyzed the relationship between the number of rearrangement events and phylogenetic proximity of genomes. Phylogenetic proximity or distance between genomes was assessed based on comparison of core genomes by calculating the number of base substitutions per site between sequences applying the Tamura–Nei model [33]. The relationship between the number of rearrangement events and the phylogenetic proximity of genomes for all studied genomes of *N. gonorrhoeae* (pairwise comparison of all 146 genomes) and *N. meningitidis* (pairwise comparison of all 138 genomes) is shown in Figure 3a,b. For the plots shown in Figure 3, the calculated values of the Spearman ranking correlation coefficient were ϱ = 0.329 (*p* < 0.001) for *N. gonorrhoeae* and ϱ = 0.624 (*p* < 0.001) for *N. meningitidis*.

Thus, the correlation between the number of rearrangement events and the phylogenetic proximity of genomes were higher for the *N. meningitidis* population than for the *N. gonorrhoeae* population. It can be assumed that the dependence of the order of genes on phylogenetic proximity is observed only at a sufficient level of nucleotide diversity in the population, i.e., at sufficiently large phylogenetic distances between isolates. The higher nucleotide diversity of the *N. meningitidis* population compared to *N. gonorrhoeae* may, in turn, mean that *N. meningitidis* is more distant from their common ancestor.

### 2.5. Rearrangements in Genomes of N. gonorrhoeae and N. meningitidis and Coordinates of Mobile Elements

Since the aim of the study was to typify and characterize the recombination points that led to the formation of syntenic blocks and to determine the causes of recombination in the past, we considered not only whole mobile elements, but also their fragments. For instance, IS elements were taken into account if their length was at least 150 bp, and phage elements if their length was at least 25% of their reference sequence (see below).

When analyzing the genome sequences of our sample, we found Correia elements (CREEs) among the mobile elements that could contribute to genomic rearrangements: on average, 126 CREEs per genome for *N. gonorrhoeae* and 250 for *N. meningitidis* (Table 1), which corresponds to the literature data [8,10,14,15]. In *N. gonorrhoeae* isolates, we identified Spencer-Smith elements with a length of about 650 bp long (three elements per genome). For *N. gonorrhoeae*, 80 NIMEs per genome were detected, for *N. meningitidis*, 288 NIMEs (Table 1).

We also identified the sequences of IS elements belonging to the IS5, IS30, IS110, and IS1595 groups (Table 1). The IS30 elements (ssgr IS1655) encoding the DDE-type transposase were found only in *N. meningitidis*, confirming the previous data about this type of IS element [20]. The IS110 elements which encode a DEDD-type transposase were found in both *Neisseria* species. The identified elements of the IS1595 group belong only to the IS1016 subgroup encoding a DDEK-type transposase. The detected elements of the IS5 group (DDE-type transposase) belong to several subgroups: IS5, IS427, etc. 

The presence of a number of prophages, both complete and fragments, was detected. Table 2 shows the sum of the lengths of phage elements when using different thresholds for their detection.

At a detection threshold of 25% or more, Neisserial filamentous phages Nf1, Nf1-MDA, Nf2; all five known phages with double-stranded DNA NgoΦ1–NgoΦ5; and all four known single-stranded (filamentous) phages NgoΦ6–NgoΦ9 were identified in the chromosomal DNA sequences of *N. gonorrhoeae*. The Nf1-MDA phage contains a meningococcal disease-associated (MDA) island, the presence of which correlates with hyperinvasive forms of gonorrhea in adults [12,22].

The chromosome of *N. meningitidis* was found to contain Nf1, Nf1-MDA, Nf2, and Nf3 single-stranded phages; NgoΦ7–NgoΦ9 single-stranded phages; and Mu-like Pnm1, Pnm2, and MuMenB phages.

**Figure 4:**
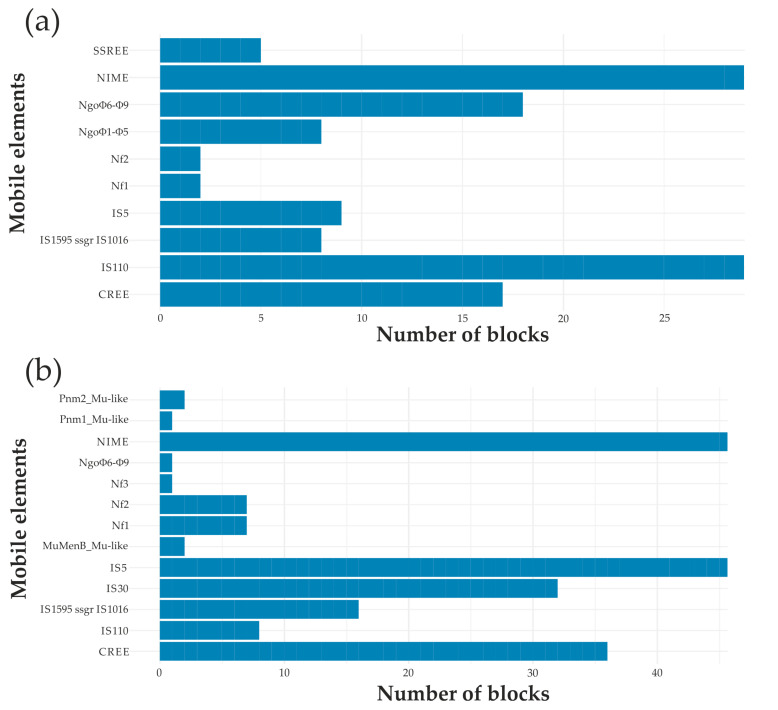
Syntenic blocks flanked by mobile elements in *N. gonorrhoeae* (**a**) and *N. meningitidis* (**b**).

The coordinates of syntenic blocks obtained using the SibeliaZ program were compared with the coordinates of mobile elements, thus determining which mobile elements could take part in the observed genomic rearrangements. The minimum distance from the block boundary to the beginning or end of the reading frame of the mobile element was considered to be no more than 700 bp for NIMEs, IS elements, and prophages, and no more than 1800 bp for CREEs and SSREEs. The lists of *N. gonorrhoeae* and *N. meningitidis* syntenic blocks for which mobile elements flanking them were found are given in Table S3. Diagrams reflecting the number of different syntenic blocks flanked by mobile elements in *N. gonorrhoeae* and *N. meningitidis* are shown in Figure 4a,b.

For *N. gonorrhoeae*, the total number of syntenic blocks was 220, out of which 97 (44.1%) were flanked by various mobile elements and prophages. For *N. meningitidis*, out of 327 syntenic blocks found, 158 (48.3%) were flanked by mobile elements and prophages. Thus, it was shown that more than 44% of genomic rearrangements in both bacterial species may have occurred with the participation of such mobile elements.

For both *N. gonorrhoeae* and *N. meningitidis*, the largest number of genomic rearrangements was associated with the presence of NIMEs; CREE and IS elements also played an important role (Figure 4). In *N. gonorrhoeae*, a significant proportion of rearrangements can be explained by the activity of single- and double-stranded phages NgoФ1–NgoФ9, the presence of large fragments (>25%) of which was found at the sites of rearrangements. The rearrangements in the *N. gonorrhoeae* genomes with the participation of Spencer-Smith elements (SSREEs are absent in *N. meningitidis*) and rearrangements in *N. meningitidis* with the participation of Mu-like prophages (absent in *N. gonorrhoeae*) should also be noted. As for IS elements, the IS110 elements were the most important for the rearrangements in *N. gonorrhoeae*, and the IS5 and IS30 elements for that in *N. meningitidis*.

Using the results of whole genome sequencing of 50 *N. gonorrhoeae* isolates collected in Russia, we analyzed the genomic rearrangements observed within the Russian *N. gonorrhoeae* population. The Wilcoxon rank-sum test allowed us to reveal that the average number of mobile elements per genome is the same both for the population as a whole and for the Russian isolates. The analysis showed that, in terms of rearrangement events, there is no difference between the Russian and global populations of *N. gonorrhoeae*.

## 3. Discussion

In this work, we studied genomic rearrangements in *N. gonorrhoeae* and *N. meningitidis* at the population level using a wide range of de novo sequenced genomes obtained from available databases as well as genomes of *N. gonorrhoeae* isolates sequenced by us. During the evolution of a microorganism, the nucleotide/amino acid sequence and the order of genes can change. It is generally accepted that the order of genes changes faster than the amino acid sequence [2,3,4,5], but the relationship between these processes has not been determined. In this work, we showed at the population level that for *N. gonorrhoeae* the statement about the interdependence of the order of genes and phylogenetic proximity is, in general, incorrect, in contrast to *N. meningitidis*, for which a stronger correlation between changes in the order of genes on the chromosome and phylogenetic proximity was found.

A strong positive correlation between rearrangement distance and amino acid distance for the genomes of 40 prokaryotes was noted by Novichkov et al. [5]. However, the distances were determined by somewhat different methods than those used by us. The distance of amino acids in [5] was characterized by the value of *dN*/*dS*, the ratio of the number of nonsynonymous (*dN*), i.e., leading to a change in the amino acid, and synonymous (*dS*) substitutions per site, while a quantitative measure of genome rearrangement was considered to be the synteny distance (*dY*) which, as noted in the article, is only a general characteristic of the evolutionary loss of the gene synteny and does not reflect specific events leading to rearrangement.

*N. meningitidis* is known to have a highly flexible and dynamic genome [20,27]. The genomes of meningococci are characterized by a large variety and diversity of repetitive DNA fragments. About 20% of its chromosome consist of repetitive sequences of all kinds [27,34]. Based on the results of our work, in which we analyzed the observed changes in synteny in the genome and compared the populations of two different *Neisseria* species, it can be assumed that the correlation of the number of rearrangements in the genome correlates with the phylogenetic distance only at a high nucleotide diversity.

For the sample of *N. gonorrhoeae* genomes, 220 syntenic blocks were identified, out of which 97 (44.1%) were flanked by various mobile elements and prophages. For the sample of *N. meningitidis* genomes, 327 syntenic blocks were identified, out of which 158 (48.3%) were flanked by mobile elements and prophages. Thus, it was shown that more than 44% of the observed rearrangements in two species of *Neisseria* are most likely to have occurred with the participation of mobile elements, including prophages. The observed variability can provide pathogens with the ability to persist in the host for a long time and re-infect them.

Analysis of transposable elements flanking syntenic blocks revealed all main mobile elements described in the literature for *N. gonorrhoeae* and *N. meningitidis* genomes that could contribute to genomic rearrangements, including prophages and their fragments. Our results showed that rearrangements most often occurred with the participation of NIMEs and IS110 in *N. gonorrhoeae* and NIMEs and IS110 and IS5 in *N. meningitidis.* Spencer-Smith elements unique for *N. gonorrhoeae* and elements belonging to IS30 group typical of *N. meningitidis* were also found. *N. gonorrhoeae* genomes contained a much large number of different phage elements as compared with *N. meningitidis.*

It should be noted that homologous recombination can occur with the participation of a large number of sequences with high homology and it is not possible to trace all of them. In addition, it is known that in *N. gonorrhoeae* recombination processes are used for antigenic variation of the pilin protein PilE [35].

Based on the results of whole genome sequencing of the Russian *N. gonorrhoeae* isolates, we analyzed the genomic rearrangements observed within the Russian population of *N. gonorrhoeae*. Despite the fact that this population differs from the European population of *N. gonorrhoeae* [30,31,36,37] in terms of isolates belonging to NG-MAST and MLST types, as well as antibiotic resistance (in particular, isolates resistant to third-generation cephalosporins have not yet been found in Russia), we did not find any differences between the Russian and the global populations both in the number of rearrangement events and in the number of mobile elements in genomes.

Modern phylogeny methods rely not only on the amino acid or nucleotide sequence data using the distance-based methods, the maximum parsimony-based methods, and the maximum likelihood-based methods, but also on the gene order data [38]. Our results on the variability of the order of genes in the considered socially significant pathogens can become the basis for creating a more sophisticated pathogen genotyping method that combines standard typing approaches, such as MLST, NG-MAST, and cgMLST, with syntenic rearrangements data.

## 4. Materials and Methods

### 4.1. Clinical Isolates of N. gonorrhoeae Collected in the Russian Federation

Clinical isolates of *N. gonorrhoeae* were collected at the State Research Center for Dermatovenerology and Cosmetology of the Ministry of Health of the Russian Federation. The samples were obtained by specialized medical organizations of the dermatovenerological profile from clinical material (urethral preparations for men and cervical/urethral preparations for women) of patients diagnosed with primary symptomatic uncomplicated gonorrhea, each sample from an individual patient. Sample collection, transportation, cultivation, and storage were performed according to a previously described protocol [36,37].

Fifty isolates obtained in 2015–2019 in 8 regions of the Russian Federation (Arkhangelsk, Astrakhan, Bryansk, Cheboksary, Kaluga, Omsk, Penza, Stavropol) were used. The isolates were grown under aseptic conditions on chocolate agar plates at 37 °C and 5% CO_2_. As a control of sequencing, the *N. gonorrhoeae* ATCC 49226 strain was used.

### 4.2. Whole Genome Sequencing of N. gonorrhoeae Isolates and De Novo Genome Assembly

Isolation of genomic DNA for sequencing was performed from an overnight culture of gonococci using the Monarch Genomic DNA Purification Kit (New England Biolabs, Ipswich, MA, USA). The resulting DNA preparations were further purified using Agencourt AMPure XP beads (Beckman Coulter, Brea, CA, USA). Final DNA concentrations were measured using a NanoDrop 2000 spectrophotometer and a Qubit 4 Fluorometer (Thermo Fisher Scientific, Waltham, MA, USA); the concentrations were in the range of 20–100 ng/µL.

Whole genome sequencing was performed in parallel on two platforms: FLO-MIN110 R9 and R10 sequencing cells in a MinION device (Oxford Nanopore Technologies, Oxford, UK) and a MiniSeq sequencing system (Illumina, San Diego, CA, USA). For the MinION cell, a library of DNA fragments was prepared using the Oxford Nanopore and New England Biolabs reagent kits according to the Native barcoding genomic DNA protocol (with EXP-NBD104, EXP-NBD114, and SQK-LSK109). According to the manufacturer’s protocol, the DNA ends were repaired with subsequent barcoding and adapter ligation. The final library (5–50 fmol DNA) was loaded onto the flow cell. For sequencing on the Illumina platform, libraries were prepared using the DNA fragmentation method followed by PCR and indexing according to the Nextera XT DNA Library Prep Kit Reference Guide (Illumina, USA). After purification of the libraries, verification of the lengths and concentrations was carried out using automated capillary electrophoresis platforms TapeStation 4150 (Agilent Technologies, Santa Clara, CA, USA). The final library was normalized and denatured according to the MiniSeq system instructions. The cluster density averaged 170–250 K/mm^2^ in all runs. Data output averaged 10.5 GB of data out of 12 GB theoretically possible on this device.

In total, ~1 GB of raw data per sample in the fastQ format was generated for each platform. Hybrid de novo genome assembly after sequencing on two platforms was carried out using the Unicycler program (https://github.com/rrwick/Unicycler, accessed on 30 June 2022). All sequences were uploaded to the GenBank, Bioproject PRJNA768989 (www.ncbi.nlm.nih.gov/bioproject/?term=PRJNA768989, accessed on 4 October 2022), under assigned accession numbers (Table S1).

To consider the possibility of genome changes during the experiment, two *N. gonorrhoeae* DNA samples were re-sequenced and re-analyzed (Bioproject PRJNA768989, SAMN31014989, SAMN22599488, SAMN31015000 and SAMN22600865). The samples were taken from the cell culture after different storage times and after reseeding the culture on a plate.

### 4.3. Genomes from the Databases

Genome sequences of isolates of the *Neisseria* genus were taken from the GenBank and BV-BRC databases (bv-brc.org). A total of 95 *N. gonorrhoeae* and 138 *N. meningitidis* genomes were found that were obtained by de novo assembly with long reads and high coverage, and assembly level—complete genome or chromosome. The genomes used in this study are listed in Table S1 of the Supplementary Materials.

### 4.4. Construction of the Phylogenetic Network

The selected genomes of *Neisseria* spp. were processed using the Prokka program v.1.14.6 (https://github.com/tseemann/prokka, accessed on 1 August 2022) to obtain .gff files with annotated genomes. The .gff files were then analyzed by rapid large-scale prokaryotic pan-genome analysis using the Roary software v.3.13.0 [39]. After the Roary processing, core genomes containing aligned sequences of concatenated orthologs were obtained, and a phylogenetic network was constructed using the SplitsTree v.5.0.0_alpha program [40].

### 4.5. Assessment of Nucleotide Diversity for N. gonorrhoeae and N. meningitidis

The value of π, the average number of pairwise nucleotide differences among genome sequences, was calculated in the Matlab program by the measurement of the Jukes–Cantor pairwise distances after realigning each pair of sequences, while ignoring the sites with gaps.

### 4.6. Calculation of the Distance between Genomes

The distance between genomes on the basis of core genomes, i.e., the number of base substitutions per site from between sequences, was calculated for 146 nucleotide sequences of *N. gonorrhoeae* (95 genomes from the database and 50 genomes of the Russian isolates, that we obtained in this work) and 138 nucleotide sequences of *N. meningitidis*. Analyses were conducted using the Tamura–Nei model [33]. Evolutionary analyses were carried out using the MEGA software v.11 [41].

### 4.7. Determination of the Minimum Number of Changes in the Structure of One Genome Compared to Other Genomes

When studying genomic rearrangements, we determined the minimum number of changes in the genome structure that allow a genome with one order of syntenic blocks to turn into a genome with a different order of syntenic blocks due to events such as insertions, deletions, inversions, translocations, fusion and fission of blocks.

The chromosomal sequences of the *N. gonorrhoeae* and *N. meningitidis* isolates from our sample (.*gbk* files) were normalized in such a way that the sequences began with the *dnaA* gene and were converted to the .*fasta* format. In the next step, we used the SibeliaZ v.1.2.5 algorithm (https://github.com/medvedevgroup/SibeliaZ, accessed on September 30 2022) [42] to build locally collinear blocks. Default algorithm parameters were used, except for *k = 15* (15 is recommended by the program developers for bacteria, 25 for mammals).

The resulting .*maf* file was then processed by the maf2synteny script available from the SibeliaZ repository. As an input parameter *b* which determines the minimum length of the syntenic block, we used the value of 1248 bp, which corresponds to the 3rd quartile (75%) of the distribution of all *N. gonorrhoeae* and *N. meningitidis* genes by length (gene sequences were taken from the PubMLST website). A smaller threshold value introduces errors, showing the presence of changes within genes, while a larger value deals with syntenic blocks of a larger size, in which rearrangement events for individual genes are not taken into account. The files blocks_coords.txt and genomes_permutations.txt were obtained, with coordinates of syntenic blocks and permutation matrices, respectively.

The minimum number of events that allow one genome to turn into another was calculated using the DCJ–indel model in the UniMoG program v.1.0 [43] (https://bibiserv.cebitec.uni-bielefeld.de/dcj, accessed on October 7 2022).

### 4.8. Determining Coordinates of Mobile Elements

Correia elements. To identify Correia elements, we used an approach described earlier in [44]. Correia elements are flanked on the right and left sides by relatively conservative 26 bp sequences, the so-called inverted Correia repeats [16]. We used the fuzznuc utility from the EMBOSS package v.6.6 (http://emboss.toulouse.inra.fr/cgi-bin/emboss/fuzznuc, accessed on October 7 2022), to the input of which the following four sequences were supplied

5′-TATAGTGGATTAACAAAAACCGGTACGG-3′,

5′-TATAGTGGATTAAATTTAAACCGGTACGG-3′,

5′-TATAGTGGATTAACAAAAATCAGGACAA-3′,

5′-TATAGTGGATTAAATTTAAATCAGGACAA-3′,

with a maximum of three mismatches allowed. The found coordinates of the inverted repeats were matched with each other in Microsoft Excel, obtaining the coordinates of the Correia elements.

Spencer-Smith elements. To identify Spencer-Smith elements, we used 19 bp repeats 5′-CGTTTCAGACGGCATCGGG-3′ and 5′-CCCGATGCCGCCTGAAACG-3′ [16]. In addition, to confirm the coordinates, we used the blast2seq utility from NCBI.

Neisserial intergenic mosaic elements (NIMEs). First, we used the fuzznuc utility to search for the flanking dRS3 sequences (5′-ATTCCCNNNNNNNGGGAAT-3′), then matched them in Excel so that they were ≤200 bp apart, and assembled a unified NIME sequence. Sequences located at a greater distance were discarded.

IS elements. To identify IS elements, we used the IS-Finder site (http://issaga.biotoul.fr, accessed on October 10 2022); 284 files in the *.xlsx* format were obtained. The files were pooled and sorted by the “*similarity_pp1*” column, discarding all IS element predictions with less than 80% similarity and/or if they were too short (<150 bp) and/or they were flagged as “*probable false positive*”.

Prophage sequences were first searched using the *PhiSpy* v.4.2.21 algorithm (https://github.com/linsalrob/PhiSpy, accessed on 14 October 2022) as described in [45], with *default* settings to find rough coordinates. To refine the coordinates, the *blastall* program from the NCBI was used, to the input of which the custom database we built was supplied. This database included the sequences of both known gonococcal and meningococcal phages: NgoΦ1–NgoΦ9 (*N. gonorrhoeae* strain FA1090), Nf1, Nf3 (*N. meningitidis* strain Z2491), Nf2 (*N. meningitidis* strain alpha710), Pnm1, Pnm2 (*N. meningitidis* strain Z2491), MuMenB (*N. gonorrhoeae* strain MC58).

The obtained coordinates of the mobile elements were compared with the coordinates of the syntenic blocks, determined as described in Section 4.7.

### 4.9. Statistical Data Processing: Correlation Analysis

The Spearman’s rank correlation test with precomputed null distribution was performed in the pspearman package v.0.3–1 of the R software v.4.2.1. The Wilcoxon Rank Sum and Signed Rank Tests were performed in the R Package stats v.4.2.1.

## 5. Conclusions

Fifty genomes of Russian *N. gonorrhoeae* clinical isolates belonging to the genogroups most common in the Russian Federation were sequenced with long reads and high coverage. All sequences were uploaded to GenBank, Bioproject PRJNA768989. De novo genome assembly allowed us to analyze the order of genes on the *N. gonorrhoeae* chromosome and to carry out the analysis of genomic rearrangements.

Population-level analysis of *N. gonorrhoeae* and *N. meningitidis* isolates revealed a stronger correlation between changes in the gene order and phylogenetic proximity for *N. meningitidis* in contrast to *N. gonorrhoeae*. This can be explained by a higher genetic diversity of *N. meningitidis* as compared with *N. gonorrhoeae.*

More than 44% of the observed rearrangements in *N. gonorrhoeae* and *N. meningitidis* genomes most likely occur with the participation of mobile elements, including prophages.

No differences were found between the Russian and global *N. gonorrhoeae* population, both in terms of rearrangement events and in the number of transposable elements in genomes.

## Figures and Tables

**Table 1:** Number of mobile elements in genomes of *N. gonorrhoeae* and *N. meningitidis* (average number of elements per genome).

Mobile Element		*N. gonorrhoeae*	*N. meningitidis*
Correia elements (CREEs)		126	250
Spencer-Smith elements (SSREEs)		3	0
Neisserial intergenic mosaic elements (NIMEs)		80	288
IS elements:	IS30 ssgr IS1655	0	12
	IS110	10	6
	IS5 ssgr IS5	1	3
	IS5 ssgr IS427	0	7
	IS5 (all sub-groups)	6	22
	IS1595 ssgr IS1016	17	12

**Table 2:** Phage elements found in *N. gonorrhoeae* and *N. meningitidis* at different detection thresholds (cut-offs). Due to the high homology between filamentous phages, the Blast algorithm assigned the same fragment to several phages.

Prophage	Sum of Lengths of Phage Elements			
	*N. gonorrhoeae*	*N. meningitidis*	*N. gonorrhoeae*	*N. meningitidis*
Nf1	43%	98%	91%	113%
Nf1-MDA	42%	112%	86%	116%
Nf2	196%	72%	214%	94%
Nf3	0%	6%	0%	14%
NgoФ1	64%	0%	130%	5%
NgoФ2	40%	0%	138%	4%
NgoФ3	88%	0%	165%	1%
NgoФ3 (another fragment)	92%	1%	149%	17%
NgoФ4	97%	0%	143%	19%
NgoФ5	79%	2%	231%	82%
NgoФ6	230%	5%	328%	48%
NgoФ7	291%	43%	408%	80%
NgoФ8	290%	43%	407%	80%
NgoФ9	230%	44%	278%	51%
Pnm1 (Mu-like)	0%	7%	0%	16%
Pnm2 (Mu-like)	0%	8%	9%	67%
MuMenB (Mu-like)	0%	15%	11%	89%
Cut-off value	>25%		>0.5%	

## Data Availability

Assembled genomes are available from the National Center for Biotechnology Information (NCBI) under BioProject accession number PRJNA768989.

## Supplementary Materials

The following supporting information can be downloaded at: https://www.mdpi.com/article/10.3390/ijms232415644/s1.
